# Decaboran: MAK-Begründung, Nachtrag

**DOI:** 10.34865/mb1770241d11_1ad

**Published:** 2026-03-30

**Authors:** Andrea Hartwig, MAK Commission

**Affiliations:** 1 Institut für Angewandte Biowissenschaften, Abteilung Lebensmittelchemie und Toxikologie, Karlsruher Institut für Technologie (KIT) Adenauerring 20a, Geb. 50.41 76131 Karlsruhe Deutschland; 2 Ständige Senatskommission zur Prüfung gesundheitsschädlicher Arbeitsstoffe, Deutsche Forschungsgemeinschaft, Kennedyallee 40, 53175 Bonn, Deutschland. Weitere Informationen: Ständige Senatskommission zur Prüfung gesundheitsschädlicher Arbeitsstoffe | DFG

**Keywords:** Decaboran, Toxizität, Reizwirkung, zentrales Nervensystem, Hautresorption, Luft, air

## Abstract

The German Senate Commission for the Investigation of Health Hazards of Chemical Compounds in the Work Area (MAK Commission) has re-evaluated the data for decaborane [17702-41-9] considering all toxicological end points. Relevant studies were identified from a literature search. Decaborane is a highly toxic substance for which limited toxicological data are available. Accidental exposure of humans resulted in effects on the nervous system such as headaches, tremor, impaired coordination, confusion, anxiety, photophobia and other symptoms. Repeated inhalation studies in animals found lethality at the lowest concentration tested of 0.05 ml/m^3^ after continuous exposure for 90 days or at 4.5 ml/m^3^ after intermittent exposure for up to 6 months. Data suitable for deriving an occupational exposure limit value (maximum concentration at the workplace, MAK value) are not available. Therefore, decaborane has been listed in Section II b of the List of MAK and BAT Values. Dermal exposure of rabbits to 10 mg/kg body weight was lethal within 7 days. Decaborane remains designated with an “H” (for substances which can be absorbed through the skin in toxicologically relevant amounts). There are no data available to evaluate the sensitizing, genotoxic, carcinogenic or reproductive toxic effects of decaborane.

**Table:** 

**MAK-Wert**	**nicht festgelegt, vgl. Abschnitt II b der MAK- und BAT-Werte-Liste**
**Spitzenbegrenzung**	**–**
	
**Hautresorption (1974)**	**H**
**Sensibilisierende Wirkung**	**–**
**Krebserzeugende Wirkung**	**–**
**Fruchtschädigende Wirkung**	**–**
**Keimzellmutagene Wirkung**	**–**
	
Synonyma	Borhydrid (B_10_H_14_) Decabortetradecahydrid
Chemische Bezeichnung (IUPAC-Name)	Decaboran
CAS-Nr.	17702-41-9
Formel	B_10_H_14_
Molmasse	122,22 g/mol
Schmelzpunkt	99,7 °C (Sivaev 2023)
Dampfdruck bei 25 °C	0,067 hPa (NCBI 2025)
Dichte bei 25 °C	0,94 g/cm^3^ (NCBI 2025)
log K_OW_	k. A.
Löslichkeit	geringfügig in kaltem Wasser, löslich in Alkohol, Benzol, Tetrachlormethan, Essigsäure (NCBI 2025)
pKs-Wert	in wässrigem Ethanol 2,41–3,21 je nach Wassergehalt (Sivaev 2023)
**1 ml/m^3^ (ppm) ≙ 5,071 mg/m^3^**	**1 mg/m^3^ ≙ 0,197 ml/m^3^ (ppm)**
	
Hydrolysestabilität	hydrolysiert in heißem Wasser (NCBI 2025)
Verwendung	zur Herstellung von Carboranen, Borbeschichtungen, Nanopartikeln, Mikrokristallen, Bornitrid-Nanoschichten und -röhren, dünnschichtigen Metallborid-Filmen, als Brennstoff für Brennstoffzellen (Sivaev 2023)
	als Katalysator für die Polymerisation von Olefinen, Gummi-Vulkanisator, bei der Herstellung von Plastik, als Sauerstofffänger (NCBI 2025)

Es liegt eine Begründung aus dem Jahr 1974 vor mit einem MAK-Wert von 0,05 ml/m^3^, der 1958 vom TLV übernommen wurde (Henschler 1974).

Ein REACH-Dossier zu Decaboran ist nicht vorhanden. Decaboran wird jedoch als Ausgangsstoff für die Synthese einiger Carborane und vielflächiger Borhydride verwendet (Sivaev 2023).

Decaboran ist ein sehr starkes Reduktionsmittel und ein farbloser, an der Luft stabiler, leicht sublimierbarer, unangenehm riechender kristalliner Feststoff. Es tritt eine Geruchs-Gewöhnung ein. Nach dem zweiten Weltkrieg wurde in den USA die Nutzung von Boran-basierten Flugzeug- und Raketenbrennstoffen erwogen, dieser Einsatz wurde jedoch wieder verworfen (Sivaev 2023).

## 1 Allgemeiner Wirkungscharakter

Decaboran wird inhalativ und dermal gut resorbiert und ist stark toxisch.

Bei 20 °C kann durch Verdampfen eine gesundheitsschädigende Konzentration auftreten, die unterhalb der Geruchsschwelle liegt. Dampf und Aerosol wirken reizend an Augen und Atemwegen. Beim Menschen kann die wiederholte Aufnahme direkte und auch verzögert eintretende Wirkungen auf das zentrale Nervensystem mit Müdigkeit, Konzentrations- oder Koordinationsstörungen sowie Übererregbarkeit und Narkose haben. Bereits kurze Expositionen gegen geringe Konzentrationen können zu permanenten Schäden der Leber führen.

Eine 90-tägige kontinuierliche Exposition gegen 0,047 ml/m^3^ ist für Affen, Ratten und Mäuse letal. Bei intermittierender Exposition gegen 4,5 ml/m^3^ sind alle Kaninchen innerhalb von einer Woche, alle Hunde innerhalb von zwei Wochen, alle Mäuse innerhalb von 19 Wochen gestorben und nur Ratten haben diese Konzentration mit leicht vermindertem Körpergewicht sechs Monate lang überlebt.

Decaboran führt zu einem Absinken des Blutdrucks und der Herztätigkeit. Es hemmt Stoffwechselvorgänge und manche Enzyme, schädigt Hirnzellen und bei wiederholter Gabe Leber und Nieren. Decaboran beschleunigt die Blutgerinnung und stört die Nukleinsäuresynthese.

Decaboran führt nach i.p. Gabe bei anästhesierten Katzen und Kaninchen zu verlangsamtem und unregelmäßigem Herzschlag. Generell zeigt sich zuerst eine Übererregung und dann eine anhaltende Depression des Nervensystems.

Es liegen keine Daten zur sensibilisierenden, reproduktionstoxischen, genotoxischen und kanzerogenen Wirkung vor.

## 2 Wirkungsmechanismus 

Decaboran ist reizend an Augen und Atemwegen.

Der Wirkungsmechanismus der hohen Toxizität von Decaboran ist unbekannt. Es gibt Hinweise auf eine Hemmung der Aspartataminotransferase (Henschler 1974) und eine Reduktion von Pyridoxalphosphat durch in wässrigem Milieu gebildete stark reduzierende Zwischenprodukte. Letztere hemmen die Aspartataminotransferase-Aktivität (Naeger und Leibman 1972). Die Zwischenprodukte wurden bisher nicht weiter identifiziert.

Bei Ratten wurde nach Gabe von 15 oder 30 mg Decaboran/kg KG mittels Fluoreszenzanalyse eine Depletion von Noradrenalin in adrenergen Nerven der Iris beobachtet, wohingegen bei Tieren, deren Sympathikusnerv durchtrennt und dadurch die Iris denerviert wurde, eine deutlich geringere Depletion auftrat (Malmfors und von Euler 1971).

Decaboran führt im Tierversuch zu einem Absinken des Noradrenalinspiegels, des Histamingehaltes im Gehirn, des Blutdrucks, der Herztätigkeit und hemmt zelluläre Stoffwechselvorgänge und schädigt vor allem Hirnzellen und bei wiederholter Gabe Leber und Nieren. Decaboran beschleunigt die Blutgerinnung, stört die Nukleinsäuresynthese und hemmt die L-Aminosäuredecarboxylase (Henschler 1974).

## 3 Toxikokinetik und Metabolismus

### 3.1 Aufnahme, Verteilung, Ausscheidung

Decaboran wird bei Inhalation und dermaler Gabe gut resorbiert (Henschler 1974).

Bei einmaliger Gabe von 15 mg Decaboran/kg KG an sechs Mischlingshunde betrugen 15, 45, 60 und 90–120 Minuten nach der Verabreichung sowie fünf Stunden bis eine Stunde vor dem Tod die Decaboran-Konzentrationen im Serum 0,85; 1; 1,5 bis 3; 2,4 bis 3,8 bzw. 4,0 bis 4,8 µg/ml (Miller et al. 1960).

Wiederholte Gabe kleiner Dosen führten zu einer kumulierten Toxizität (siehe Abschnitt 5.2; Svirbely 1955).

### 3.2 Metabolismus

Im wässrigen Milieu wird Decaboran schnell zu nicht identifizierten polaren Intermediaten bzw. stark reduzierenden Zwischenprodukten umgesetzt. Im Organismus erfolgt möglicherweise eine Umsetzung bis zur Borsäure (Naeger und Leibman 1972).

## 4 Erfahrungen beim Menschen

Es liegen keine Daten zur genotoxischen, reproduktionstoxischen, kanzerogenen oder haut- und atemwegssensibilisierenden Wirkung von Decaboran vor.

Die Geruchsschwelle wird mit 0,3 mg/m^3^ angegeben und der Geruch wird als schokoladenartig und Übelkeit erregend beschrieben (Henschler 1974). Bei Ruth (1986) wird eine Geruchsschwelle von 0,36 mg/m^3^ genannt. Vermutlich gehen die Angaben auf die Untersuchung von Comstock und Oberst (1953) zurück, in der eine Geruchsschwelle von 0,35 mg/m^3^ (0,07 ml/m^3^, im Bericht fälschlich als 0,7 ml/m^3^ angegeben) an Probanden bestimmt wurde.

Ein Zwanzigjähriger reparierte und wusch eine Pumpe, die Decaboran enthielt. Dabei zog er im Korridor vor dem Pumpenraum ca. 20- bis 30-mal seine Maske aus und registrierte den Geruch „eines Gases“. Beim abschließenden Säubern der Pumpe bemerkte er leichten Schwindel, fühlte sich jedoch wohl, bis er zu Hause nach dem Abendessen weiche Knie und wiederum Schwindel bekam. Er schlief die Nacht geräuschvoll und nach dem Aufstehen waren Schwindel und weiche Knie noch immer da. Er hatte keine Sehstörungen, Kopfschmerzen oder Übelkeit. Während der Fahrt zur Arbeit fühlte er sich müde, die Untersuchungen im Unternehmen ergaben jedoch nichts Ungewöhnliches. Dennoch fuhr er nach Hause und schlief drei Stunden am Nachmittag und 13 Stunden in der Nacht. Am Folgetag wollte er zur Arbeit fahren, hatte aber Koordinationsstörungen und kehrte um. Er wurde ins Krankenhaus gebracht und auf dem Weg traten unwillkürliche Kontraktionen der Nackenmuskeln auf, sodass der Kopf sich nach rechts und links drehte. Seine rechte Körperseite versteifte sich. Bei der Fahrt im Rollstuhl lehnte er sich über die linke Seite, im Bett war sein Gesicht gerötet und er schüttelte sich wie bei Schüttelfrost und klagte über Schmerzen. Es wurde ihm Phenobarbital verabreicht, was ihn etwas beruhigte. Bei der physischen Untersuchung waren die Nacken- und Rückenmuskeln versteift und der rechte Arm und das rechte Bein zitterten. Augen, Nase, Mund, Brust, Herz waren unauffällig, die Bauchmuskeln verhärtet und der Rand der Leber konnte nicht ertastet werden. Nach einiger Zeit traten schwerer Tremor und Kontraktionen der Arme auf, die Hände waren geballt, der Unterarm gebeugt und er schwitzte stark. Nach einer Gabe von Meperidin entspannte er sich deutlich innerhalb einiger Minuten. Am nächsten Tag ging es ihm besser, jedoch waren Thymol-Trübung (Test auf erhöhte beta- und gamma-Globuline) und Kephalin-Cholesterin-Flockung (Leberfunktionstests) erhöht. Acht Tage nach der Exposition wurde der Patient entlassen, ohne dass sich die Tests auf Thymol-Trübung und Kephalin-Flockung verändert hatten. Spätere Untersuchungen sind nicht berichtet (Lowe und Freeman 1957).

Weitere 19 exponierte Arbeiter mit milden Symptomen berichteten vor allem von Übelkeit und Kopfschmerzen (k. w. A.; Lowe und Freeman 1957).

In den 1950er und 1960er Jahren waren über 2000 Menschen bei der Herstellung von Borhydriden mittels eines Pyrolyseprozesses beschäftigt. Durch Unfälle wurde die stark toxische Wirkung von Borhydriden und ihrer Derivate bekannt. Bei Exposition gegen Decaboran kam es zu Kopfschmerzen, Tremor, unkoordinierten Bewegungen, Konfusion, Ängstlichkeit, Lichtempfindlichkeit und anderen Symptomen. Bereits geringe Konzentrationen auch unterhalb der Geruchsschwelle führten zu Vergiftungserscheinungen. Es tritt jedoch eine Gewöhnung bei der Geruchswahrnehmung auf. Eine wiederholte Exposition gegen Decaboran kann zu schweren Nervenschädigungen führen (k. w. A.) (Sivaev 2023).

Bei 20** °**C kann durch Verdampfen von Decaboran eine gesundheitsschädigende Konzentration entstehen (k. w. A.). Es führt mit Latenzzeiten bis zu 48 Stunden zu Konzentrationsschwäche, Verwirrtheit, Übelkeit, Kopfschmerzen, feinschlägigem Tremor, intermittierenden Kontraktionen der Nacken- und Extremitätenmuskulatur, Konvulsionen, mäßiger bis mittelschwerer Schädigung der Leberfunktion und tubulären Nierenschäden. Todesfälle sind nicht bekannt geworden (Henschler 1974).

## 5 Tierexperimentelle Befunde und In-vitro-Untersuchungen

### 5.1 Akute Toxizität

#### 5.1.1 Inhalative Aufnahme

Es wurden je zehn männliche, nicht gefastete CFW-Mäuse oder je fünf männliche, nicht gefastete CFW-Ratten vier Stunden lang gegen Decaboran (Reinheit > 98 %) in Konzentrationen von 17,2; 24,2; 30,7; 37,3; 47,7 (nur Mäuse); 60,7; 77,2 oder 95,2 ml/m^3^ exponiert. Zur Erzeugung der Expositionskonzentrationen wurde Luft durch Kammern mit definierter Temperatur mit Decaboran-Kristallen geleitet. Die Konzentrationen wurden aus der evaporierten Menge an Decaboran berechnet. Die LC_50_ betrug für Mäuse bezogen auf 24 und 48 Stunden Nachbeobachtung 36,5 bzw. 25,7 ml/m^3^. Da keine der Ratten innerhalb von zwei Wochen nach der Exposition starb, lag die LC_50_ für Ratten in dieser Untersuchung deutlich oberhalb von 95,2 ml/m^3^. Die Mäuse waren unruhig, schwach, zeigten schnelle und angestrengte Atmung, hatten Hornhauttrübungen und waren auch am nächsten Tag noch träge. Die Ratten hatten auch Hornhauttrübungen und leichten Nasenausfluss und zeigten zum Teil leichte Gleichgewichtsprobleme (Svirbely 1954 a).

#### 5.1.2 Orale Aufnahme

Die orale LD_50_ jeweils bei 48 Stunden Nachbeobachtung betrug bei Ratten 64,3 mg/kg KG und bei Mäusen 40,9 mg/kg KG (Henschler 1974).

#### 5.1.3 Dermale Aufnahme

Die LD_50_ bei Ratten betrug bei perkutaner Gabe 740 mg/kg KG bei 48 Stunden Nachbeobachtung (Svirbely 1955) und 640 mg/kg KG (k. w. A.; Roush 1959). Für Kaninchen lag die perkutane LD_50_ nach 48 Stunden bei 71 mg/kg KG und nach 24 Stunden bei 113 mg/kg KG (Svirbely 1955). Bei Kaninchen traten zentralnervösen Störungen und Leberschäden auf (Henschler 1974).

#### 5.1.4 Intraperitoneale Aufnahme 

Die LD_50_ bei i.p. Gabe an Ratten betrug 27 mg/kg KG (k. w. A.; Roush 1959). Bei 48 Stunden Nachbeobachtung war sie bei Ratten 23,4 mg/kg KG und bei Mäusen 33 mg/kg KG (Henschler 1974).

Bei einmaliger Gabe von 15 mg Decaboran/kg KG an sechs Hunde trat ein Zusammenhang zwischen der Konzentration an Decaboran im Serum und der Toxizität auf. Eingesetzt wurde Decaboran von hoher Reinheit (k. w. A.) in Olivenöl. Die ersten 30 Minuten nach der Gabe zeigten die Tiere keine Anzeichen einer toxischen Wirkung. Decaboran war 15 Minuten nach der i.p. Gabe im Serum nachweisbar und erreichte eine Konzentration von 0,85 µg/ml bevor Anzeichen von Toxizität auftraten. Nach ca. 45 Minuten erreichte der Serumspiegel eine Konzentration von 1 µg/ml und die Tiere wurden ängstlich, erbrachen sich und litten unter unregelmäßigem Zittern. Innerhalb von einer Stunde erreichte die Borankonzentration 1,5 bis 3 µg/ml und die Tiere zitterten und hatten unkoordinierte Muskelbewegungen. Es traten 90 bis 120 Minuten nach der Applikation Krämpfe mit zunehmender Intensität auf. Jeder Anfall dauerte 60 bis 90 Sekunden und wurde teilweise durch Atemstillstand unterbrochen. In diesem Stadium lag der Serumspiegel von Decaboran bei 2,4 bis 3,8 µg/ml. Die Tiere erhielten keine Medikamente gegen die Krämpfe und wurden fünf Stunden bis eine Stunde vor dem Tod komatös. In dieser Zeitspanne lag der Serumspiegel von Decaboran bei 4,0 bis 4,8 µg/ml (Miller et al. 1960**)**.

### 5.2 Subakute, subchronische und chronische Toxizität

#### 5.2.1 Inhalative Aufnahme

Die Studien zur wiederholten inhalativen Aufnahme sind in Tabelle 1 dargestellt.

Es wurden zehn männliche CFW-Ratten (bereits mehrere Monate im Laboratorium gehalten) und sechs männliche Meerschweinchen (heterogene Gruppe, von einem Züchter erworben) sechs Stunden pro Tag, an 21 Tagen innerhalb von 32 Tagen gegen Decaboran (Reinheit > 98 %) exponiert. Es wurden je fünf männliche Kontrolltiere mitgeführt. Zur Erzeugung der Expositionskonzentrationen wurde Luft durch Kammern mit definierter Temperatur mit Decaboran-Kristallen geleitet und die Masse an verdampftem Decaboran durch die Menge an durchgeleiteter Luft dividiert. Die berechnete Expositionskonzentration betrug 20 ml/m^3^. Die exponierten Ratten verloren während der Expositionstage an Gewicht und erholten sich etwas an expositionsfreien Tagen. Zum Expositionsende hatten die exponierten Ratten im Vergleich zu Studienbeginn ca. 75 g an Gewicht verloren und waren erst ca. drei Wochen nach Expositionsende wieder auf dem Ausgangsgewicht (Ablesung aus Abbildung 1, keine Zahlenangaben). Der Gewichtsverlust wurde mit einem möglichen Appetitverlust durch die Exposition gegen Decaboran erklärt. Die klinischen Symptome verstärkten sich mit den Expositionstagen und verringerten sich auch durch expositionsfreie Zeiten nicht. Es traten verschiedene Effekte wie Ataxie, Zuckungen und Krämpfe sowie leichter Ausfluss aus den Augen und der Nase auf, mit zunehmender Exposition wurden die Befunde stärker. Auch eine Woche nach Expositionsende waren Apathie, Zuckungen im Gesicht, Augenblinzeln, Reizbarkeit und Nervosität zu beobachten. Es wurden hämatologische Untersuchungen vor, während und nach der vierwöchigen Expositionsphase durchgeführt, wofür die Probennahme am Morgen erfolgte, also ca. 16 Stunden nach einer Exposition. Die Erythrozytenzahl und der Hämatokrit waren während der Exposition erhöht, was nach Angaben der Autoren eventuell auf Dehydratisierung durch Gewichtsverlust zurückzuführen ist. Nach der Exposition waren Hämatokrit und Hämoglobin statistisch signifikant verringert. Die Zahl an polymorphkernigen Leukozyten während und nach der Expositionsperiode war signifikant erhöht. Die Untersuchung von zwei Ratten nach zweiwöchiger Exposition zeigte vergrößerte Nebennieren, leichte Fettleber und Abmagerung. Nach dreiwöchiger Exposition waren diese Befunde bei drei weiteren untersuchten Tieren verstärkt, die Nebennieren hatten zudem dunkle Punkte, die Leber verstopfte und verfettete Stellen, wobei die Lungen normal aussahen, die Schnittstellen jedoch verstärkt bluteten. Am Ende der Expositionszeit von 21 Tagen wurden noch vier Tiere untersucht, die stark abgemagert waren. Sie hatten vergrößerte Nebennieren mit vielen dunklen Stellen, die Leber war bröckelig oder verfettet. Die Lungen erschienen normal. Bei den Kontrolltieren wurden keine Auffälligkeiten der Organe beobachtet. Gegen 20,9 ml/m^3^ exponierte Meerschweinchen mussten nach fünf bis sechs Tagen moribund getötet werden oder starben mit massivem Gewichtsverlust. Die Wirkung von Decaboran scheint sich zu akkumulieren, da die Toxizität bei wiederholter Exposition massiver ist als bei akuter Exposition (Svirbely 1954 b).

Sprague-Dawley-Ratten, Swiss-Mäuse und Rhesus-Affen wurden 90 Tage lang kontinuierlich gegen eine mittlere Konzentration von 0,047 ml/m^3^ exponiert. Die Reinigung der Käfige und Versorgung der Tiere mit Futter und Wasser dauerte täglich ca. 34–46 Minuten, in denen die Konzentration im Käfig um ca. 50 % sank. Es dauerte ca. 60 Minuten, bis die Ziel-Konzentration wieder erreicht war. Das Körpergewicht wurde alle 14 Tage bestimmt und jeder Affe einzeln gewogen, während bei Ratten (n = 5) und Mäusen (n = 10) ein Gesamtgewicht pro Käfig ermittelt wurde. Histopathologisch untersucht wurden Leber, Nieren, Lunge, Herz, Pankreas, Milz, Nebennieren, Rückenmark sowie Cortex und Medulla des ZNS aller Affen und von je 40 % der Ratten und Mäuse. Dabei bestand die 40 %-Auswahl zur einen Hälfte aus während des Versuches gestorbenen und zur anderen Hälfte aus überlebenden Tieren. Drei Wochen nach Expositionsbeginn waren alle Tiere geschwächt und zitterten teilweise. Sie wurden immer schwächer bis zu ihrem Tod. Nach sechs und elf Wochen kamen Krämpfe hinzu und die Atmung war schwer. Die Mortalität betrug bei Affen 60 %, bei Ratten 64 % und bei Mäusen 82 %. Die Befunde sind in Tabelle 1 aufgeführt (House 1964). Es wurde nur eine Konzentration mit kontinuierlicher Exposition untersucht, die für Mäuse, Ratten und Affen toxisch und letal war. Daher kann aus dieser Untersuchung nicht auf eine Konzentration ohne adversen Effekt (NOAEC) geschlossen werden.

Wenn Labortiere 5,5 bis 6 Stunden am Tag, an fünf Tagen pro Woche für einen Zeitraum von sechs Monaten gegen 4,5 ml Decaboran/m^3^ exponiert waren, reagierten Kaninchen am empfindlichsten, da nach zwei Tagen alle fünf Tiere gestorben waren. Hunde (alle drei Tiere nach vier Tagen gestorben) und Affen (nur 13 Tage exponiert, zu diesem Zeitpunkt waren zwei von drei Tieren gestorben) waren weniger empfindlich. Nach 19 Wochen waren alle (k. w. A.) Mäuse gestorben. Nach sechs Monaten waren die einzigen Befunde bei Ratten ein vermindertes Körpergewicht, verminderter Hämatokritwert und eine verminderte Zahl an weißen Blutzellen. Alle Tiere überlebten. Erste Anzeichen von Toxizität bei Hunden und Affen waren periorbitale Entzündung, Erbrechen, Anorexie, fortschreitende Schwäche, Verweigerung von Fressen und Verlust der muskulären Koordination. Histopathologische Veränderungen zeigten sich bei den Tieren in der zentralen Zone der Leber und den Nierentubuli. Bei Hunden traten zusätzlich Lungenödem (Henschler 1974; House 1964) und Hyperplasie des Knochenmarks (House 1964) auf. Es kann aus dieser Untersuchung nicht auf eine NOAEC geschlossen werden.

**Tab. 1:** Toxizität von Decaboran nach wiederholter inhalativer Exposition

**Spezies,** **Stamm, ** **Anzahl pro Gruppe**	**Exposition**	**Befunde^a)^**	**Literatur**
**Ratte**, CFW, 10 ♂ exponiert, 5 ♂ Kontrolle	**4 Wo**, 0, 20 ml/m^3^, 6 h/d, 5 d/Wo außer Feiertagen, 21 Expositionen in 32 Tagen, Ganzkörper, Reinheit > 98 %	**20 ml/m^3^**: Nervosität, Teilnahmslosigkeit, Kauerhaltung, Augenblinzeln, Unruhe, Atemnot, Ataxie, Schluckauf-ähnliche Krämpfe, Zuckungen im Gesicht, unkoordinierte Muskeln, Zucken und Schütteln des Kopfes, leichte Krämpfe, leichter Ausfluss aus Augen u. Nase, mit der Zeit zunehmende Krämpfe u. Schreckhaftigkeit, KG ↓ (33. Tag ca. 75 g KG-Abnahme), Erythrozytenzahl, Hämatokrit- und Hämoglobinwert verändert (s. Text), Zahl polymorphkerniger Leukozyten ↑, ab 3. Wo: Nebennieren vergrößert z. T. mit dunklen Stellen, Leber z. T. gestaut, Fetteinlagerungen, ein Tier mit Ödem in Lunge, ein anderes mit gestauten Stellen in Lunge; nach einer Woche Nachbeobachtung: Apathie, Zuckungen im Gesicht, Augenblinzeln, Reizbarkeit, Nervosität	Svirbely 1954 b
**Ratte**, Sprague Dawley, je 50 ♂, KG: 260–290 g	**90 d**, 0; 0,047 ml/m^3^ (MW), Konzentrationsbereich: 0,005–0,17 ml/m^3^, 24 h/d, 7 d/Wo, 40 % der Tiere histopathologisch untersucht (s. Text), KG/Käfig (5 Tiere) alle 14 d bestimmt	**0,047 ml/m^3^**: Mortalität 64 %, Tiere wurden immer schwächer, geschwollenes Gesicht, Futter- u. Wasseraufnahme ↓, KG-Zunahme pro Käfig ↓, Zittern, Krämpfe, Erythrozytenzahl ↑, Hämatokrit ↑, Stresstest: Schwimmzeit ↓, 11 Tiere Bronchopneumonie, 11 Tiere Bronchiektasen, Peribronchitis, 7 Tiere Nephritis, 1 Tier Vakuolen in renalen Tubuli, 1 Tier fokale Nekrose Leber	House 1964
**Ratten**, k. w. A.	**6 Mo**, 4,5 ml/m^3^, 5–6 h/d, 5 d/Wo	KG leicht ↓, Hämatokritwert ↓, Zahl weißer Blutzellen ↓, keine Mortalität, k. w. A.	Henschler 1974; House 1964
**Maus**, ICR Swiss, je 100 ♂, KG: ca. 30 g	**90 d**, 0; 0,047 ml/m^3 ^(MW), Konzentrationsbereich: 0,005–0,17 ml/m^3^, 24 h/d, 7 d/Wo, 40 % der Tiere histopathologisch untersucht (s. Text), KG/Käfig (10 Tiere) alle 14 d bestimmt	**0,047 ml/m^3^**: Mortalität 82 %, Futter- u. Wasseraufnahme ↓, Tiere wurden immer schwächer, Zittern, Krämpfe, Stresstest: Schwimmzeit ↓, 12 Tiere Makrophageninfiltration in Lunge, 7 Tiere erhöhte bronchiale Sekretion, 20 Tiere polymorphkernige Leukozyteninfiltration Lunge, 13 Tiere Peribronchitis, 5 Tiere Bronchopneumonie, 2 Tiere Fettablagerung in Nebennieren, 3 Tiere Vakuolen in tubulärem Nierenepithel	House 1964
**Maus**, k. w. A.	**19 Wo**, 4,5 ml/m^3^, 5–6 h/d, 5 d/Wo	keine spezifischen toxischen Symptome, bis 19. Wo: 100 % Mortalität	Henschler 1974; House 1964
**Affe**, 3, k. w. A.	**2 Wo**, 4,5 ml/m^3^, 5–6 h/d, 5 d/Wo (13 Expositionen), alle Tiere histopathologisch untersucht	periorbitale Entzündung, Erbrechen, Anorexie, fortschreitende Schwäche, Verweigerung von Fressen, Verlust der muskulären Koordination, Mortalität: 2/3 Tiere, Reizung an Augen u. Nase, leichte ZNS-Symptome, Leber- u. Nierenschäden	Henschler 1974; House 1964
**Affe**, Rhesus, je 10 ♂, KG: ca. 3 kg	**90 d** , 0; 0,047 ml/m^3 ^(MW), Konzentrationsbereich: 0,005–0,17 ml/m^3^, 24 h/d, 7 d/Wo	**0,047 ml/m^3^**: Mortalität 60 %, einige Tage Durchfall um 30. Tag, Tiere wurden immer schwächer, geschwollenes Gesicht (Überlebende), Zittern, Krämpfe, schwere Atmung, Stresstest: Schwimmzeit unverändert, gestaute Leber mit Fetteinlagerung, z. T. fokale Nekrose in Leber, Kalzifizierung in Leber, Nieren mit fettigen Veränderungen, Nekrosen im proximalen Tubulus, Kristalle in Nieren, Ausdehnung im rechten Ventrikel des Herzens, z. T. fokale Degeneration und myocarditische Stellen im Herzen, Stau in Milz und Nebennieren, 1 Tier Kalzifizierung im Cortex des ZNS, 1 Tier im Rückenmark, Befunde in Lunge wegen Lungenmilben bei exponierten und Kontrolltieren	House 1964
**Hund**, 3, k. w. A.	**4 d**, 4,5 ml/m^3^, 5–6 h/d	periorbitale Entzündung, Erbrechen, Anorexie, fortschreitende Schwäche, Verweigerung von Fressen und Verlust der muskulären Koordination, Mortalität 2/3 nach 3. Tag, ein Tier am 4. Tag moribund getötet, schwere ZNS-Symptome, Leber u. Nieren geschädigt, Lungenödem, Hypoplasie des Knochenmarks	Henschler 1974; House 1964
**Kaninchen**, 5, k. w. A.	**2 d**, 4,5 ml/m^3^, 5–6 h/d	Mortalität: 2/5 am 1. Tag, 3/5 am 2. Tag, Organe makroskopisch ohne Befund	Henschler 1974; House 1964
**Meerschweinchen**, „heterogene Gruppe“, 6 ♂, KG: 700–900 g	**5 d**, 20,9 ml/m^3^, 6 h/d, 5–6 d da moribund, Ganzkörper	**20,9 ml/m^3^**: Atemnot, entzündete oder opalisierte Augen, farbloser Ausfluss aus Augen, Trägheit, Krämpfe, Lustlosigkeit, Tendenz auf rechte Seite zu lehnen, gekrümmte Haltung, Zittern, Mortalität: 1 nach 5. Tag (314 g KG verloren) u. 4 moribund getötet (189–221 g KG verloren), 1 nach 6. Tag (211 g KG verloren), gestaute Leber mit Fetteinlagerungen u. Pigmentierung, Lungen klar oder gestaut mit leichten Ödemen	Svirbely 1954 b

^a)^ wenn nicht anders angegeben, sind die aufgeführten Veränderungen statistisch signifikant

d: Tag; h: Stunde; KG: Körpergewicht; Konz.: Konzentration; Mo: Monat; MW: Mittelwert; Wo: Woche; ZNS: zentrales Nervensystem

**Fazit:** Bei der wiederholten Exposition gegen 4,5 ml Decaboran/m^3^ war die Reihenfolge der Empfindlichkeit anhand der Mortalität Kaninchen > Hund > Affe > Maus > Ratte (keine Mortalität) (House 1964).

#### 5.2.2 Orale Aufnahme

Decaboran (Reinheit > 98 %) wurde als 0,25%ige Agar-Salzlösung sieben Tage lang oral (k. w. A.) an je fünf männliche CFW-Ratten pro Gruppe verabreicht. Bei Gabe von 10 mg/kg KG und Tag starben zwei Tiere nach der siebten Gabe. Bei 20 mg/kg KG starben zwei Tiere am dritten Tag, eines am vierten und eines am fünften Applikationstag. Bei 40 mg/kg KG starben zwei Tiere am zweiten und die restlichen Tiere am dritten Applikationstag. Es wurden Nervosität, Übererregbarkeit, Atemnot, Ataxie, Krämpfe, Zittern, schlaffe Lähmung, Koma und massiver Gewichtsverlust beobachtet. Bei den die siebentägige Gabe überlebenden Tieren wurde eine Erholung zwei Wochen nach der letzten Substanzgabe beobachtet, wobei diese Tiere noch immer ein vermindertes Gewicht aufwiesen. Bei den gestorbenen Tieren wurden Fettlebern, gestaute Lungen mit Anzeichen von Ödemen und Gas im Intestinaltrakt nachgewiesen (Svirbely 1955). Aus dieser Untersuchung ergibt sich kein NOAEL.

Nach oraler Gabe von 3 mg Decaboran/kg KG und Tag in Gelatine-Kapseln an drei Mischlingshündinnen starb je ein Tier nach 10 und 46 Stunden, das dritte Tier wurde 26 Stunden nach der fünften Dosis moribund getötet. Es traten Erbrechen, Anorexie, Teilnahmslosigkeit, Dilatation der konjunktivalen Blutgefäße über der Sklera, Nervosität, erhöhte Atemfrequenz, Ataxie, rhythmisches Zittern des Kopfes, beschleunigter Puls, verlangsamte Atmung, verlangsamter Herzschlag und Mortalität auf. Noch 18 Stunden nach der dritten Substanzgabe zeigte sich eine erhöhte Farbstoffretention in der Leber und ein Anstieg an polymorphkernigen Neutrophilen im Blut. Im Urin war der Kreatininspiegel erhöht, kurz vor dem Tod fanden sich auch Blut und Albumin im Urin. Bei der Autopsie war die Leber entweder leicht gestaut oder bleich und fettig, die Milz gestaut, das Pankreas pinkfarben und die Lungen ohne Befund (Svirbely 1955). Aus dieser Untersuchung ergibt sich kein NOAEL.

Im Vergleich zur akuten Gabe trat bei wiederholter Gabe von Decaboran die Mortalität bei deutlich niedrigeren Konzentrationen bzw. Dosierungen auf. Der Autor schließt auf eine kumulative Wirkung (Svirbely 1955).

#### 5.2.3 Dermale Aufnahme

Um die perkutane Aufnahme von Decaboran an je fünf männlichen Ratten oder drei männlichen Neuseeländer-Kaninchen pro Gruppe zu untersuchen, wurde der Feststoff in einem minimalen Volumen an Dioxan gelöst, dem Maiskeimöl zugemischt war, und auf die geschorene intakte Rückenhaut aufgetragen. Bei 37,5 mg/kg KG und Tag starb eine Ratte nach der fünften, eine nach der sechsten Dosis, die anderen beiden Tiere überlebten sieben Applikationen. Bei Verabreichung von 75 mg/kg KG starben alle fünf Ratten nach der dritten Gabe, bei Verabreichung von 150 mg/kg KG starben vier Tiere nach der zweiten und das letzte Tier nach der dritten Dosierung. Von den Kaninchen starb ein Tier nach der dritten Dosis von 5 mg/kg KG und Tag, die beiden anderen überlebten eine siebentägige Gabe. Wurden 10 mg/kg KG und Tag appliziert, starb ein Kaninchen nach der zweiten Dosis, ein weiteres nach der sechsten Dosis und das dritte am siebten Tag. Bei Applikation von 25 mg/kg KG und Tag waren alle drei Kaninchen nach drei Tagen gestorben. Die Anzeichen für Toxizität bei den Ratten waren denen nach intraperitonealer Gabe ähnlich. Zusätzlich zeigten die Kaninchen Opisthotonus, Katarakte verschiedener Schweregrade, keine Reaktion auf leichte Stimulation bzw. ungewöhnliche Reaktion bei Stimulation. Eine Regeneration wurde bei den überlebenden Kaninchen auch eine Woche nach der letzten Applikation nicht beobachtet. Bei Ratten und Kaninchen traten keine Anzeichen von Reizwirkung an der Applikationsstelle und als histopathologische Veränderungen ausschließlich kleine Areale von Leberverfettung auf (Svirbely 1955). Aus dieser Untersuchung ergibt sich kein NOAEL.

#### 5.2.4 Intraperitoneale Aufnahme

Decaboran (Reinheit > 98 %) wurde als 0,25%ige Agar-Salzlösung intraperitoneal an je fünf männliche CFW-Ratten pro Gruppe verabreicht. Bei Gabe von 5 mg/kg KG starb ein Tier nach der siebten Dosierung, ein weiteres innerhalb der anschließenden siebentägigen Nachbeobachtung. Bei Gabe von 10 mg/kg KG starb ein Tier nach der zweiten, zwei nach der dritten und zwei nach der vierten Dosis. Bei Gabe von 20 mg/kg KG starb ein Tier nach der ersten Dosis, die restlichen Tiere nach der zweiten Dosis. Anzeichen für Toxizität waren nach siebentägiger Gabe auch am zwölften Tag danach noch Nervosität, Erregbarkeit, Zuckungen im Gesicht, krampfartige Bewegungen, unkoordinierte Bewegungen, entzündete Augen und verminderte Gewichtszunahme. Es wurden keine histopathologischen Veränderungen gefunden. Zwei Kaninchen erhielten einmal täglich 1 mg Decaboran/kg KG. Eines starb nach der vierten, das andere nach der sechsten Substanzgabe. Wurden 2 mg/kg KG an vier Kaninchen verabreicht, starb eines nach der dritten, zwei nach der fünften und eines nach der sechsten Dosierung. Es traten nur geringfügige zentralnervöse Effekte auf (k. w. A.) (Svirbely 1955). Aus dieser Untersuchung ergibt sich kein NOAEL.

Es wurde die Toxizität von Decaboran und dessen Derivat HEF-3 (High Energy Fuel 3, Ethyldecaboran) von hoher Reinheit (k. w. A.) in Olivenöl an Mischlingshunden, Neuseeländer-Kaninchen und Rhesus-Affen untersucht. Es erfolgten i.p. Gaben um toxische, subtoxische und nicht toxische Dosierungen zu ermitteln. Für die Ermittlung des nicht toxischen Bereiches wurde je drei Hunden und je drei Kaninchen einen Monat lang täglich 25, 100 oder 250 µg Decaboran/kg KG verabreicht. Die Ermittlung der Blutkonzentration erfolgte wöchentlich, 24 Stunden nach der letzten Gabe wurde die Decaborankonzentration in Gehirn und Leber untersucht. Es traten keine klinischen Symptome auf und es ließ sich kein Decaboran im Serum nachweisen. Bei 100 und 250 µg/kg KG wurden 0,1 bis 0,32 µg Decaboran/g Gewebe in Leber und Gehirn gefunden (Miller et al. 1960**)**. Da keine histopathologischen Untersuchungen erfolgten, kann diese Untersuchung nur eingeschränkt zur Bewertung herangezogen werden.

Intraperitoneal verabreichtes Decaboran (k. w. A.) verlangsamte bei anästhesierten Katzen und Kaninchen den Herzschlag und machte ihn sehr unregelmäßig. Atropin, Epinephrin, Ephedrin, Dextroamphetamin oder alpha-Hypophamin hatten darauf keinen Einfluss (Krackow 1953). Generell zeigt sich zuerst eine Übererregung und dann eine Depression des Nervensystems, die Stunden anhalten konnte (Cole et al. 1954).

Die i.p. Gabe von 1 mg/kg KG an aufeinander folgenden Tagen an zwölf Mischlingshunde, zwölf Neuseeländer-Kaninchen und sechs Rhesus-Affen erfolgte so lange, bis die Tiere Anzeichen von Toxizität zeigten. Beobachtet wurden nur Effekte auf das zentrale Nervensystem wie verminderte motorische Aktivität, vermindertes Interesse an der Umgebung und ein stumpfsinniger oder depressiver Zustand. Kaninchen zeigten bis zum 6. Tag keine, ab dem 9. Tag Symptome. Hunde zeigten fünf Tage lang keinen Effekt, aber bei siebentägiger Gabe eine ZNS-Depression. Eine Aussage zu den Affen ist nicht möglich, da sie lernten „sich krank zu stellen“, auch wenn ihnen nur eine physiologische Kochsalzlösung verabreicht wurde (Miller et al. 1960).

### 5.3 Wirkung auf Haut und Schleimhäute

#### 5.3.1 Haut

Hierzu liegen keine spezifischen Untersuchungen vor.

Wurde Decaboran in einem minimalen Volumen an Dioxan gelöst, dem Maiskeimöl zugemischt war, wiederholt dermal auf die geschorene Rückenhaut von Ratten und Kaninchen verabreicht, so traten bei siebentägiger Gabe keine Befunde an der Applikationsstelle auf (Svirbely 1955).

#### 5.3.2 Auge

Hierzu liegen keine spezifischen Untersuchungen vor.

Wurden Meerschweinchen fünf Tage lang, sechs Stunden pro Tag, gegen 20,9 ml Decaboran/m^3^ exponiert, wurden entzündete oder opalisierte Augen und farbloser Ausfluss aus den Augen beschrieben. Ähnliches wurde bei Ratten beobachtet, die vier Wochen lang an sechs Stunden pro Tag, fünf Tage pro Woche gegen 20 ml Decaboran/m^3^ exponiert waren: Augenblinzeln sowie leichter Ausfluss aus Augen und Nase (Svirbely 1954 b). Auch bei Affen, die 13 Expositionen an fünf Tagen pro Woche, sechs Stunden pro Tag gegen 4,5 ml Decaboran/m^3^ erhielten, kam es zu Reizungen an Augen und Nase (Henschler 1974; House 1964).

Diese Untersuchungen zeigen eine reizende Wirkung von Decaboran-Dämpfen am Auge.

### 5.4 Allergene Wirkung

Es liegen keine Daten oder Hinweise zur haut- und atemwegssensibilisierenden Wirkung von Decaboran vor.

### 5.5 Reproduktionstoxizität

Hierzu liegen keine Untersuchungen vor.

### 5.6 Genotoxizität

Hierzu liegen keine Untersuchungen vor.

### 5.7 Kanzerogenität

Hierzu liegen keine Untersuchungen vor.

## 6 Bewertung

Das grundlegende Vorgehen zur Bewertung eines Arbeitsstoffes ist der MAK- und BAT-Werte-Liste zu entnehmen (DFG 2025). Der kritische Effekt von Decaboran ist seine hohe Toxizität und letale Wirkung.

**MAK-Wert. **Aus Arbeitsunfällen ist bekannt, dass Decaboran beim Menschen auf das Nervensystem wirkt und u. a. zu Kopfschmerzen, Tremor, unkoordinierten Bewegungen, Konfusion oder Ängstlichkeit führen kann (Sivaev 2023). Es wurden dabei jedoch keine Expositionsmessungen in der Luft durchgeführt, sodass aus diesen Daten kein MAK-Wert abgeleitet werden kann.

In einer 90-Tage-Inhalationsstudie aus dem Jahr 1964 mit kontinuierlicher Exposition gegen 0,05 ml Decaboran/m^3^, der einzigen untersuchten Konzentration, starben über 60 % der Affen, Ratten und Mäuse (House 1964). Bei Exposition an sechs Stunden pro Tag, fünf Tage die Woche gegen 4,5 ml Decaboran/m^3^ starben alle fünf Kaninchen und drei Hunde innerhalb von vier Tagen und alle Mäuse bis zur 19. Woche, nur Ratten überlebten diese Expositionshöhe sechs Monate lang mit leicht vermindertem Körpergewicht (Henschler 1974; House 1964).

Die Substanz ist extrem toxisch, daher müsste ein Grenzwert für den Arbeitsplatz deutlich unterhalb des bisherigen MAK-Wertes von 0,05 ml/m^3^ liegen. Da sich aus den vorliegenden Untersuchungen aufgrund aufgetretener Mortalität keine NOAEC ableiten lässt, kann kein MAK-Wert aufgestellt werden und Decaboran wird dem Abschnitt II b der MAK- und BAT-Werte-Liste zugeordnet. Eine Spitzenbegrenzung entfällt.

**Fruchtschädigende Wirkung. **Da kein MAK-Wert abgeleitet wird, entfällt die Zuordnung zu einer risikobasierten Schwangerschaftsgruppe. Es liegen keine Daten zur Entwicklungstoxizität und auch keine Daten vor, aus denen sich ein Verdacht auf eine solche Wirkung ergibt.

**Krebserzeugende Wirkung. **Es liegen keine Kanzerogenitätsstudien und keine Hinweise auf eine solche Wirkung vor. Es erfolgt keine Einstufung von Decaboran in eine der Kategorien für Kanzerogene.

**Keimzellmutagene Wirkung. **Es liegen keine Untersuchungen zur Gentoxizität und kein Strukturverdacht vor. Es erfolgt keine Einstufung von Decaboran in eine der Kategorien für Keimzellmutagene.

**Hautresorption. **Es liegen keine Untersuchungen zur dermalen Penetrationsrate vor.

Die mit Kaninchen bestimmte dermale 24-Stunden-LD_50_ betrug 113 mg/kg KG. Bei wiederholter dermaler Applikation waren 5 und 10 mg/kg KG für Kaninchen letal, für Ratten 37,5 mg/kg KG (Abschnitt 5.1.3 und 5.2.3). Bei Ratten und Kaninchen traten keine Anzeichen von Reizwirkung an der Applikationsstelle und als histopathologische Veränderungen ausschließlich kleine Areale von Leberverfettung auf (Svirbely 1955).

Decaboran besitzt eine hohe Toxizität, die sich bei dermaler Gabe an Ratten und Kaninchen mit LD_50_-Werten deutlich unterhalb von 1000 mg/kg KG zeigt.

Die Markierung mit „H“ wird beibehalten.

**Sensibilisierende Wirkung. **Zur sensibilisierenden Wirkung von Decaboran liegen keine Befunde beim Menschen und keine Ergebnisse aus experimentellen Untersuchungen am Tier oder aus Untersuchungen aus alternativen Testverfahren (NAMs) vor. Decaboran wird daher weder mit „Sh“ noch mit „Sa“ markiert.
